# PACC: Large scale connected component computation on Hadoop and Spark

**DOI:** 10.1371/journal.pone.0229936

**Published:** 2020-03-18

**Authors:** Ha-Myung Park, Namyong Park, Sung-Hyon Myaeng, U Kang

**Affiliations:** 1 Kookmin University, Seoul, Republic of Korea; 2 Carnegie Mellon University, Pittsburgh, PA, United States of America; 3 KAIST, Daejeon, Republic of Korea; 4 Seoul National University, Seoul, Republic of Korea; National Institute of Advanced Industrial Science and Technology, JAPAN

## Abstract

A connected component in a graph is a set of nodes linked to each other by paths. The problem of finding connected components has been applied to diverse graph analysis tasks such as graph partitioning, graph compression, and pattern recognition. Several distributed algorithms have been proposed to find connected components in enormous graphs. Ironically, the distributed algorithms do not scale enough due to unnecessary data IO & processing, massive intermediate data, numerous rounds of computations, and load balancing issues. In this paper, we propose a fast and scalable distributed algorithm PACC (Partition-Aware Connected Components) for connected component computation based on three key techniques: two-step processing of partitioning & computation, edge filtering, and sketching. PACC considerably shrinks the size of intermediate data, the size of input graph, and the number of rounds without suffering from load balancing issues. PACC performs 2.9 to 10.7 times faster on real-world graphs compared to the state-of-the-art MapReduce and Spark algorithms.

## Introduction

A connected component in a graph is a set of nodes linked to each other by paths. Finding connected components is a fundamental graph mining task having various applications including reachability [1, 2], pattern recognition [3, 4], graph partitioning [5, 6], random walk [7], graph compression [8, 9], etc. However, graphs of interest are enormous with billions of nodes and edges; e.g., 2.4 billion monthly active users form a huge friendship network in Facebook https://newsroom.fb.com/company-info/ and the Web is a gigantic network where more than 1 trillion Web pages are linked to each other by hyperlinks https://googleblog.blogspot.com/2008/07/we-knew-web-was-big.html. Then, how do we efficiently compute all connected components in such massive graphs?

Hadoop and Spark are the de facto standard distributed data processing frameworks composing their own ecosystem with diverse libraries. The ecosystem provides a consistent way to store, process, and analyze data so that multiple types of big-data analyses run on the same data at massive scale on commodity hardware. Several algorithms to find connected components on Hadoop and Spark have been proposed to take the ecosystem’s advantages; however, ironically, they do not scale enough due to unnecessary data IO and processing, massive intermediate data, numerous rounds, and load balancing issues.

In this paper, we propose PACC (Partition-Aware Connected Components), a fast, scalable, and distributed algorithm for computing connected components. PACC achieves high performance and scalability by three techniques: two-step processing (partitioning and computation), edge filtering, and sketching. In the two-step processing, the partitioning step divides the graph into subgraphs gradually in several rounds so that the subgraphs can be processed independently of each other in the computation step. PACC distributes workloads evenly to machines, and avoids ‘the curse of the last reducer’ issue [10], which the most advanced MapReduce algorithm [11] suffers from. We found that, during the partitioning step, most edges arrive early in subgraphs where they should be located eventually. Our proposed edge filtering shrinks the size of intermediate data by excluding every edge that settles down in a subgraph in each iteration. Note that, the edge filtering is due to the two-step processing, and thus, it is not applicable to previous MapReduce algorithms. We also found that even if we replace a subgraph of the input graph with another subgraph that has the same connectivity, the connectivity of the input graph does not change. The sketching reduces the size of the input graph by performing a sequential connected component algorithm on each subgraph of the input graph. This paper makes the following contributions:

- **Algorithm**. We propose PACC, a fast and scalable algorithm for connected component computation in an enormous graph. PACC is made up of three key techniques: two-step processing (partitioning and computation), edge filtering, and sketching. The techniques make PACC distribute workloads evenly, shrink the size of input and intermediate data, and reduce the round number.
- **Theory**. We theoretically prove various characteristics of PACC and the correctness. We guarantee the input size never increases in every step of PACC.
- **Experiment**. We evaluate the performance of PACC using real and synthetic graphs. Experimental results show that PACC is up to 10.7 times faster than state-of-the-art MapReduce and Spark algorithms.

This paper is an extended version of [12]; in this paper, we newly propose a sketching technique that improves the performance of the previously proposed method (namely PACC-ef) by reducing the input data size. We provide the detailed proofs of the correctness and the performance of PACC with sketching (Lemmas 4, 6, 8, and 9). The efficacy of sketching is measured also experimentally; PACC with sketching shows up to 3.3 times faster performance on the graphs of Twitter and YahooWeb than PACC without sketching, i.e., PACC-ef. We describe how to implement PACC with sketching on Hadoop and Spark, and measure the impact of the systems on the performance of PACC. The datasets used in this paper and the code of PACC can be found in https://github.com/kmudmlab/PACC. Frequently used symbols are summarized in Table 1.

**Table 1:** Table of symbols.

Symbol	Definition
*G* = (*V*, *E*)	Undirected graph. *V*: node set, *E*: edge set.
*u*, *v*, *n*	Nodes.
(*u*, *v*)	Edge between *u* and *v*.
Γ(*u*)	= {*v*|(*u*, *v*) ∈ *E*} Set of neighbors of *u*.
Γ^+^(*u*)	= {*v*|*v* ∈ Γ(*u*), *v* > *u*} Set of large neighbors of *u*.
Γ^−^(*u*)	= {*v*|*v* ∈ Γ(*u*), *v* < *u*} Set of small neighbors of *u*.
*ρ*	Number of partitions.
*ξ*	Hash function *V* → {0, ⋯, *ρ* − 1}.
*ξ*(*u*)	Partition containing *u*.
[*S*]_*i*_	= {*v*|*v* ∈ *S*, *ξ*(*v*) = *i*} *i*-th partition of a set S.
*m*(*u*)	= min(Γ(*u*) ∪ {*u*}) Minimum node in Γ(*u*) ∪ {*u*}.
*m*_*i*_(*u*)	= min([Γ(*u*) ∪ {*u*}]_*i*_) Minimum node in [Γ(*u*) ∪ {*u*}]_*i*_.
*τ*	Threshold for the number of input edges.
*C*	Number of chunks.
*E*_*i*_	*i*-th chunk such that *E* = ⋃_*i*∈{0,⋯,*C*−1}_ *E*_*i*_ and *E*_*i*_ ∩ *E*_*j*_ = ∅ if *i* ≠ *j*.
*G*_*i*_ = (*V*_*i*_, *E*_*i*_)	Edge-induced subgraph of *E*_*i*_ where *V*_*i*_ is the node set.
$m_{i}^{+}\left(u\right)$	= min([Γ^+^(*u*) ∪ {*u*}]_*i*_) Minimum node in [Γ^+^(*u*) ∪ {*u*}]_*i*_.
*r*_*i*_(*u*)	Representative node of the connected component containing *u* in *G*_*i*_; the minimum node among nodes connected to *u* by a path in *G*_*i*_.
$E_{i}^{\prime }$	Graph where each node *u* in *G*_*i*_ is linked to *r*_*i*_(*u*) by an edge.
*E*′	Union of $E_{i}^{\prime }\;\forall i\in \{0,\cdots ,C-1\}$.
*G*′ = (*V*, *E*′)	Result by the first step of sketching; edge-induced subgraph of *E*′.
*G*″	Result graph of sketching.

## Related work

Connected component algorithms have been developed in different ways to deal with large scale graphs. In this section, we first introduce single-machine algorithms that the proposed algorithm can exploit as a module, and then we introduce distributed-memory and MapReduce algorithms.

### Single-machine algorithms

Breadth-first-search and depth-first-search are well known graph traversal algorithms that compute connected components in linear time. Patwary et al. [13] propose a multi-core algorithm that is based on the disjoint-set data structure; each set corresponding to a connected component and two sets are unified if an edge links nodes in the two sets. However, these algorithms cannot handle graphs exceeding the size of the main memory as they store the entire graph on the memory. GraphChi [14] and DSP-CC [15] are external algorithms that use external memory such as hard disks to increase the size of processable data in a single machine. GraphChi has an implementation for connected component computation based on iterative message passing process. DSP-CC is an external algorithm for computing connected components based on Union-Find, showing notable speed on graphs with billion nodes and edges by exploiting solid-state drives (SSDs). PACC can use these single-machine algorithms as a module, in other words, PACC can be seen as a tool that enhances single-machine algorithms to be distributed algorithms running on Hadoop and Spark.

### Distributed-memory algorithms

Many distributed connected component algorithms are proposed in a parallel random-access machine (PRAM) model, a theoretical parallel processing model. Bader and Cong [16] investigate practical algorithms implemented on symmetric multiprocessors (SMPs), and propose a SMP algorithm that first finds a shallow spanning tree of the input graph and computes connected components by conducting the depth-first search starting from the spanning tree in parallel. Several graph mining platforms that use distributed-memory such as Pregel [17], GraphLab [18], GraphX [19], PowerGraph [20], PowerLyra [21], Ligra [22], and Gemini [23] provide implementations for computing connected components that are based on iterative message passing process. To achieve high performance, they make several implicit assumptions: (1) the entire graph including replicated nodes fits into the distributed memory (Pregel, GraphLab, GraphX, PowerGraph, PowerLyra, Ligra, Gemini), (2) the set of nodes fits into the memory of a single machine (Gemini), (3) node ids are represented by 32-bit integers and consecutive (GraphLab, PowerGraph, PowerLyra, Ligra, Gemini), and (4) the input file should be in a system-specific format (Ligra, Gemini). These assumptions make the above distributed-memory algorithms not scalable, not seamlessly connected with other data processing, and require complex preprocessing.

### MapReduce algorithms

MapReduce [24] is a widely-used distributed computing framework. Exploiting distributed storage, MapReduce provides fault-tolerance of data, high scalability in terms of data and cluster sizes, and an easy-to-use interface. Thanks to the advantages of MapReduce, diverse graph mining tasks such as graph visualization [25, 26], subgraph enumeration [27], triangle counting [28, 29], and radii/diameter calculation [30] have been researched on MapReduce.

Recently, several MapReduce algorithms have been proposed for computing connected components. A naïve method to compute connected components on MapReduce is to repeat the breadth-first search (BFS) for each connected component. However, this algorithm requires too many rounds; for each connected component, the algorithm takes as many rounds as the diameter of the component. In MapReduce, the number of required rounds significantly affects overall performance. Pegasus [30] and Zones [31, 32] conduct the breadth-first search from every node concurrently so that the number of required rounds is reduced to the largest diameter of connected components in a graph. However, the number of rounds is still large for several enormous datasets; for example, the diameter of the YahooWeb graph is above 30. Hash-Greater-to-Min [33] reduces and guarantees the number of rounds to be *O*(log |*V*|) on the number of nodes for computing connected components. Hash-to-Min in [33] does not ensure that the number of round is logarithmic, but is faster than Hash-Greater-to-Min in practice. However, Hash-Greater-to-Min and Hash-to-Min generate massive intermediate data that is more than twice the original graph size, resulting in a severe performance degradation. However, both algorithms spawn large amounts of additional data during the execution process, resulting in significant performance degradation.

Two MapReduce algorithms, *two-phase* and *alternating*, proposed in [11] resolve the intermediate data explosion problem of Hash-to-Min. The algorithms guarantee that the intermediate data size of each round is always less than or equal to the input data size. However, the algorithms suffers from the load-balancing problem, which is another performance bottleneck. The alternating algorithm is introduced in more detail in the next section because it is relevant to our work.

The same paper [11] also proposes optimized algorithms of *two-phase* and *alternating*. The optimized two-phase algorithm, namely *two-phase-DHT*, exploits a distributed hash-table (DHT) to decrease the round number. The experimental result of the paper shows that the two optimized algorithms outperform the two-phase and alternating algorithms, while the optimized alternating algorithm is faster than the two-phase-DHT algorithm on graphs with above billion nodes and edges.

## Preliminaries

In this section, we introduce the definitions of a connected component and the computation of it. Then, we introduce the alternating algorithm, a MapReduce algorithm for computing connected components.

### Problem definition

A connected component is a subgraph whose nodes are connected by paths, and the formal definition is as follows.

**Definition 1** *Given an undirected graph*, *a connected component is a node set where nodes have a path to each other and have no path to a node outside the set*.

**Fig 1:**
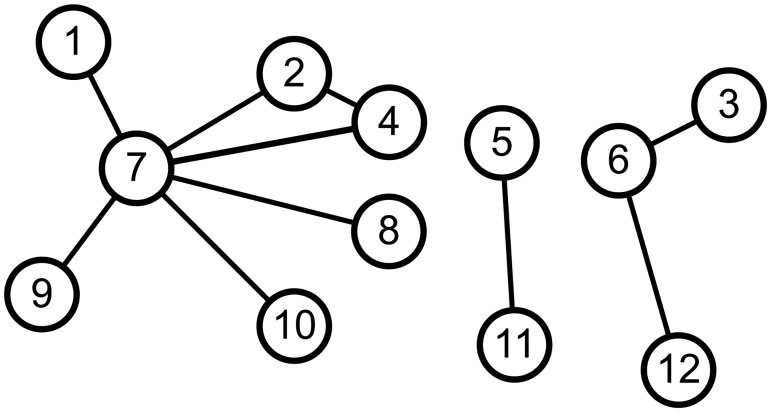
An example graph with 3 connected components.

Fig 1 is an example graph with three connected components: {5, 11}, {3, 6, 12}, and {1, 2, 4, 7, 8, 9, 10}. The computation of connected components is to detect all connected components in a graph. Equivalent task to connected component computation is to find *the minimum reachable node* from each node; the minimum reachable node from a node *u* is the node with the smallest identification number among nodes connected by a path from *u*. Note that we assume each node has a unique number and the numbers are in a total order. Each minimum reachable node represent a connected component. For example in Fig 1, nodes 1, 3, and 5 are the representatives of connected components in the graph. The following is the formal definition of the computation of connected components.

**Definition 2** *The connected component computation on a graph G* = (*V*, *E*) *is to map each node u* ∈ *V* to the minimum reachable node from node *u*.

The result of connected component computation on the example graph in Fig 1 is {(1, 1), (2, 1), (4, 1), (7, 1), (8, 1), (9, 1), (10, 1), (5, 5), (11, 5), (3, 3), (6, 3), (12, 3)}. For a node *u*, Γ(*u*) = {*v*|(*u*, *v*) ∈ *E*} is the set of *u*’s neighbors. We denote the small neighbor of *u* by Γ^−^(*u*) = {*v*|*v* ∈ Γ(*u*), *v* < *u*}. Similarly, we denote the large neighbor of *u* by Γ^+^(*u*) = Γ(*u*) \ Γ^−^(*u*). We denote by *m*(*u*) = min(Γ(*u*) ∪ {*u*}) the node that has the smallest number among *u* and *u*’s neighbors. In Fig 1, for example, Γ(7) = {1, 2, 4, 8, 9, 10}, Γ^−^(7) = {1, 2, 4}, Γ^+^(7) = {8, 9, 10}, and *m*(7) = 1.

### The alternating algorithm

The alternating algorithm is one of the two MapReduce algorithms proposed in [11]. The large-star and the small-star are the core operations of the algorithm. Each operation gets a graph as input and generates a new graph as output. The large-star operation outputs an edge (*v*, *m*(*u*)) for each *u* and for each *v* ∈ Γ^+^(*u*). Similarly, the small-star operation outputs an edge (*v*, *m*(*u*)) for each *u* and for each *v* ∈ Γ^−^(*u*) \ {*m*(*u*)}. The algorithm repeats the two operations alternately until no more edge is added or deleted.

After several rounds of the alternating algorithm, the input graph is changed into a star graph. It implies that several nodes will have a numerous neighbors while most nodes will have few neighbors. This leads to ‘the curse of the last reducer’ issue [10] meaning that most computations are performed on a small number of reducer, causing an abnormal increase in the running time of a MapReduce algorithm. In the same paper, the authors try to handle the load-balancing issue by the optimized alternating algorithm that makes several copies of high-degree nodes and spreads the high-degree nodes’ neighbors to the copies. However, the optimized alternating algorithm can increase the number of edges during the process, and the load-balancing issue still remains as we show in our experiments. Note that our method ensures that the number of edges does not increase (see Lemma 5), while resolving the load balancing problem.

## Proposed method

In this section, we propose PACC (Partition-Aware Connected Components), a fast, scalable and distributed algorithm for computing connected components. We summarize the challenges in developing a distributed algorithm for computing connected components efficient and scalable, and how PACC addresses the challenges as follows.

1. How can we resolve the load balancing problem, which the alternating algorithm suffers from? **Two-step processing of partitioning and computation** hinders edges from congregating around a few nodes; thereby, the workloads are balanced across machines.
2. How can we shrinks the input and shuffled data sizes? **Filtering out edges that are inside a partition or no longer change** rapidly decreases the number of edges every round. Consequently, the shuffled data size is also decreased. Moreover, **finding connected components on edge disjoint subgraphs of the input graph (sketching)** shrinks the number of edges in the initial graph.
3. How can we minimize the round number? As edge-filtering decreases the number of edges in each round, PACC reduces the round number by **performing a single machine computation instead of multiple rounds of distributed computation** when the edge number falls below a threshold.

In the following subsections, we first show the overview of PACC, and describe the three core techniques of PACC with theoretical analyses. Then, we discuss the issues of implementation on Hadoop and Spark.

### Overview

**Fig 2:**
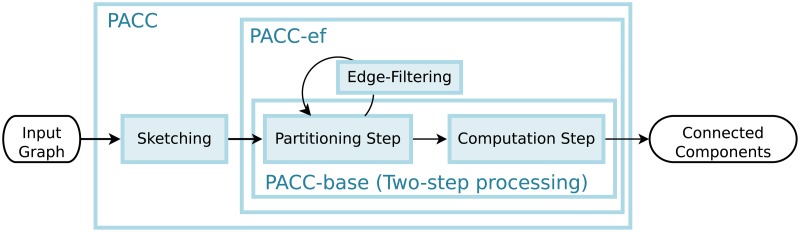
A high level overview of PACC.

Fig 2 illustrates the overview of PACC. PACC consists of two main steps (partitioning and computation) and two optimization techniques (edge-filtering and sketching). We define PACC without sketching as PACC-ef, and PACC-ef without edge-filtering as PACC-base, i.e., PACC-base consists only of the two main steps: partitioning and computation. In PACC-base, the partitioning is an iterative operation that transforms the input graph into another graph that has the same connectivity; the transformed graph is divided into *ρ* subgraphs so that the computation step correctly computes the connected components by processing each subgraph independently by a sequential algorithm. Note that subgraphs can be processed together in the same machine or independently in different machines because partitions are logical divisions of data. In PACC-ef, the edge-filtering eliminates edges that have settled on a subgraph during the partitioning step to shrink the intermediate data size for each iteration. It is worthwhile to mention that the edge-filtering is available due to the two-step processing as the partitioning step focuses only on *partitioning* rather than computing connected components; thus, the edge-filtering is not applicable to the alternating algorithm discussed in the preliminaries. In PACC, the sketching transforms the input graph into a smaller graph based on the fact that even if a portion of the graph is replaced by another graph that has the same connectivity, the transformed graph keeps the connectivity of the original graph. A similar idea is already applied to DSP-CC [15], an external algorithm, but the idea is not directly applicable to PACC because the idea is prone to load-balancing issues on distributed algorithm. The sketching of PACC carefully converts the input data to avoid load-balancing issues. The following sections describe the PACC-base, PACC-ef, and PACC in turn.

### PACC-base: Two-step processing for load-balancing

This section introduces PACC-base, a basic version of PACC without sketching and edge-filtering. We first describe the two main steps of PACC-base, and show how to combine the two steps to compute connected components.

#### The partitioning step

**Fig 3:**
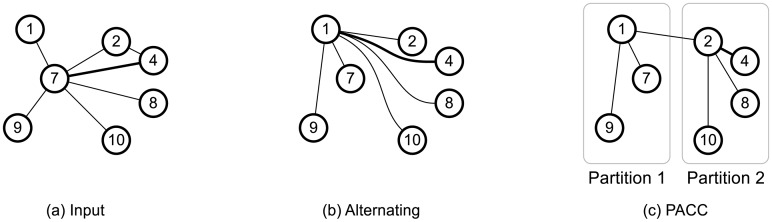
A round of the alternating algorithm and PACC transforms the graph in (a) to (b) and (c), respectively. While all edges are concentrated on node 1 by the alternating algorithm, PACC spreads the edges into *ρ* = 2 partitions.

The partitioning step of PACC-base resolves the load-balancing issue of the alternating algorithm. The key idea is to partition the nodes and to avoid connecting nodes in different partitions by edges. The main cause of the alternating algorithm’s load-balancing issue is that the edges are concentrated on a few nodes after several rounds. A round of the alternating algorithm transforms the graph in Fig 3a to the graph of Fig 3b. All edges are connected to node 1, meaning that the computation in the next round is concentrated on node 1. Meanwhile, PACC distributes the edges evenly to the partitions (see Fig 3c), resolving the load-balancing issue. PACC-base divides the nodes into *ρ* partitions using a hash function *ξ*: *V* → {0, ⋯, *ρ* − 1}. We use *ξ*(*u*) to denote the partition of a node *u*. For a subset *S* ⊆ *V* of nodes, we define the *i*-th partition of *S* by [*S*]_*i*_ = {*v* ∈ *S*|*ξ*(*v*) = *i*}.

**Fig 4:**
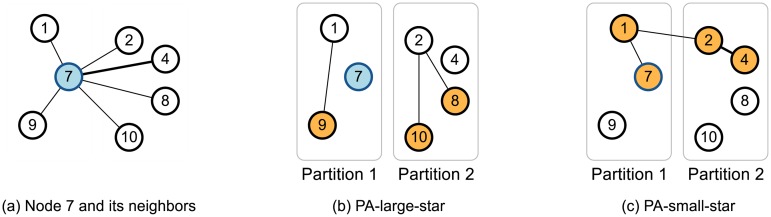
An example of the two distributed operations of PACC-base at node 7. PA-large-star links large neighbors (in orange) to the local minimum nodes. PA-small-star links small neighbors and node 7 (in orange) to the local minimum nodes. PA-small-star links node 2 to node 1 as node 2 is a local minimum node.

The partitioning step alternately performs two distributed operations, PA-large-star and PA-small-star, which improve the two core operations of the alternating algorithm by taking into account the partition of nodes. While the large-star and the small-star link the neighbors of each node *u* to the minimum neighbor *m*(*u*) of *u*, the PA-large-star and the PA-small-star link the neighbors of each node *u* to the ‘local’ minimum neighbor *m*_*i*_(*u*) = min([Γ(*u*) ∪ {*u*}]_*i*_) of *u* in the *i*-th partition, and link the local minimum neighbors *m*_*i*_(*u*) for *i* ∈ {0, ⋯, *ρ* − 1} to the ‘global’ minimum neighbor *m*(*u*). PA-large-star and PA-small-star are responsible for large neighbors Γ^+^(*u*) of *u*, and small neighbors including *u* (i.e., Γ^−^(*u*) ∪ {*u*}), respectively. Fig 4 demonstrates an example. Given a graph in Fig 4a, 4b and 4c show examples of PA-large-star and PA-small-star on node 7 when the partition number is 2. Nodes 1, 7, and 9 are in partition 1, and nodes 2, 4, 8, and 10 are in partition 2. The global minimum node *m*(7) of node 7 is node 1, and the local minimum nodes *m*_1_(7), *m*_2_(7) of node 7 are nodes 1 and 2, respectively. PA-large-star links node 9 to node 1, and nodes 8 and 10 to node 2 since large neighbors Γ^+^(7) of node 7 are nodes 8, 9, and 10. PA-small-star links node 7 to node 1, and node 4 to node 2 since small neighbors Γ^−^(7) of node 7 are nodes 1, 2, and 4. Node 2 is linked to node 1 as node 2 is a local minimum node and node 1 is the global minimum node. Algorithms 1 and 2 show how PA-large-star and PA-small-star is implemented in a distributed manner using MapReduce. Note that both PA-large-star and PA-small-star maintain the connectivity of the input graph (see Lemma 2).

**Algorithm 1**: PA-large-star

 **Map**   : input 〈*u*;*v*〉

1 emit 〈*u*;*v*〉 and 〈*v*;*u*〉

 **Reduce**  : input 〈*u*;Γ(*u*)〉

2 **foreach** *v* ∈ Γ^+^(*u*) **do**

3  **if** *v* ≠ *m*_*ξ*(*v*)_(*u*) **then**

4   emit 〈*v*;*m*_*ξ*(*v*)_(*u*)〉

5  **else if** *v* ≠ *m*(*u*) **then**

6   emit 〈*v*; *m*(*u*)〉

**Algorithm 2**: PA-small-star

 **Map**   : input 〈*u*;*v*〉 where *u* > *v*

1 emit 〈*u*;*v*〉

 **Reduce**  : input 〈*u*;Γ^−^(*u*)〉

2 **foreach** *v* ∈ Γ^−^(*u*) ∪ {*u*} **do**

3  **if** *v* ≠ *m*_*ξ*(*v*)_(*u*) **then**

4   emit 〈*v*;*m*_*ξ*(*v*)_(*u*)〉

5  **else if** *v* ≠ *m*(*u*) **then**

6   emit 〈*v*;*m*(*u*)〉

#### The computation step

The computation step divides the resulting graph of the partitioning step into *ρ* subgraphs and computes connected components in each subgraph independently. The *i*-th subgraph is the edge-induced subgraph on the edges incident to the nodes in the *i*-th partition. For example in Fig 3c, the first subgraph contains nodes 1, 7 and 9, and the second subgraph contains nodes 1, 2, 4, 8, and 10. The computation step independently computes connected components in each subgraph. The computation step is accomplished by a single round MapReduce task; the map step builds the subgraphs, and the reduce step computes connected components in the subgraphs. The map step sends each edge (*u*, *v*) to a partition *ξ*(*u*) where *u* > *v* (without lossing generality as the graph is undirected). Then, a reduce operation receives the edge set *E*_*p*_ of the *p*-th subgraph, and computes connected components from the edge-induced subgraph on *E*_*p*_ using a single machine algorithm (LocalCC). In our experiments, Union-Find with the path compression [34] is used for LocalCC. A pseudo code for the CC-Computation is in Algorithm 3.

**Algorithm 3**: CC-Computation

 **Map**   : input 〈*u*;*v*〉 where *u* > *v*

1 emit 〈*ξ*(*u*);(*u*, *v*)〉

 **Reduce**  : input 〈*p*;*E*_*p*_〉

2 LocalCC(*E*_*p*_)

Independent execution of LocalCC on each subgraph ensures to compute connected components in the original input graph. For a connected component *X*, the local minimum node min([*X*]_*i*_) in *i*-th partition is adjacent to the global minimum node min([*X*]) in the *i*-th subgraph, and the other nodes in the *i*-th partition is adjacent to min([*X*]_*i*_).

**Lemma 1**. *In the resulting graph of PACC-base’s partitioning step*, *the edge-induced subgraph on the edges incident to the nodes in a partition is a star graph where the center is the minimum node in the partition, and the star graph includes the minimum node in the connected component where the center node belongs to*.

#### Putting it together

Algorithm 4 is the pseudo code of the complete PACC-base algorithm. PACC-base performs PA-large-star and PA-small-star alternately until convergence to partition the input graph. The algorithm converges when no edges are added or deleted in a round. Then, PACC-base computes the connected components from the resulting subgraphs using the CC-Computation operation (Algorithm 3).

**Algorithm 4**: PACC-base (PACC without edge-filtering and sketching)

 **Input**: Edges (*u*, *v*) as a set *E* of key-value pairs 〈*u*;*v*〉

 **Output**: A unique connected component id for every node *v* ∈ *V*

1 *out* ← *E*

 // Partitioning step: lines 2 through 5

2 **repeat**

3  *out* ← PA-large-star(*out*)

4  *out* ← PA-small-star(*out*)

5 **until** *Convergence*;

6 **return** CC-Computation(*out*) // Computation step

### PACC-ef: Edge-Filtering

**Fig 5:**
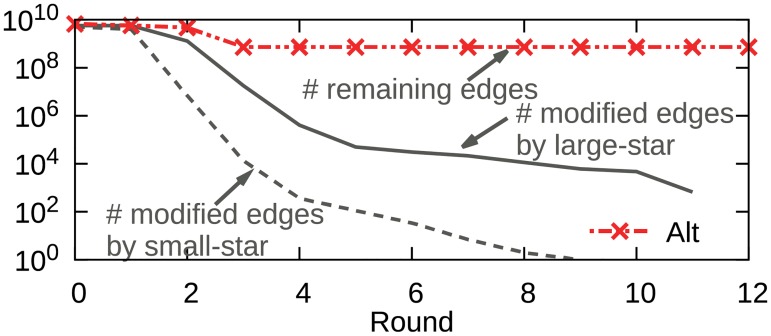
The number of input edges (in red) and modified edges (in gray) of the alternating algorithm. The number of input edges in each round does not fall below the number of non-root nodes while the number of modified edges drops sharply. This suggests that most edges are just read and written without change.

We propose PACC-ef that improves PACC-base with edge-filtering. We first show our observation that the input edge size cannot be smaller than the number of non-root nodes while the number of edges changed by the two operations of the alternating algorithm decreases rapidly every round (see Fig 5). This suggests that most edges are just read and written without change. The lower bound of the input size at each round on the alternating algorithm is proved in Theorem 1.

**Theorem 1**. *In the alternating algorithm, the number of input edges in each round does not fall below* |*V*| − |*C*| *where* |*V*| and |*C*| *are the numbers of nodes and connected components*, *respectively*.

*Proof*. The last round of the alternating algorithm outputs star graphs, each of that consists of the nodes in a connected component and the center node is the connected component’s minimum node, i.e., the output of the final round contains |*V*| − |*C*| edges. Besides, the number of output edges monotonically decreases every round by Lemmas 2 and 3 in [11]. As the input of every round except the first round is the output of the previous round, the number of input edges in a round cannot be less than |*V*| − |*C*|.

The partitioning step of PACC-ef excludes numerous edges so that the size of input and shuffled data decrease significantly every round. The edge-filtering is possible because the partitioning step focus on partitioning instead of finding connected components. PACC-ef excludes an edge (*u*, *v*) in two cases, where *u* < *v* without losing generality:

Case 1 *ξ*(*u*) = *ξ*(*v*) and Γ(*v*) = {*u*}: *u* and *v* are in the same partition, and *u* is the only neighbor of *v*.
Case 2 Γ^−^(*u*) = ∅, and Γ(*v*) = {*u*}∀*v* ∈ Γ(*u*): *u* does not have small neighbor, and every neighbor of *u* does not have neighbor except *u*.

Edges of case 1 can be excluded as the goal of the partitioning step is to divide the graph into subgraphs that can be processed independently each other during the computation step. Thereby, each filtered edge in case 1 stays in the partition, and thus, the edges filtered in case 1 form a forest graph where no edge spans partitions. Meanwhile, edges of case 2 together form star graphs, in each of them the center is the minimum node. Such edges no longer change in the subsequent rounds by PA-large-star and PA-small-star. Such edges can be excluded in the next round safely.

**Algorithm 5**: PACC-ef

 **Input**: Edges (*u*, *v*) as a set *E* of key-value pairs 〈*u*;*v*〉; a threshold number *τ* of edges to run a single machine algorithm.

 **Output**: A connected component id for each node *v* ∈ *V*

1 *out* ← *E*;*in* ← ∅;*cc* ← ∅

 // Partitioning step: lines 2 through 12

2 **repeat**

3  **if** # *edges in out* > *τ* **then**

4   (*lout*, *lcc*, *lin*) ← PA-large-star-opt(*out*)

5   *cc* ← *cc* ∪ *lcc*

6   *in* ← *in* ∪ *lin*

7   (*sout*, *sin*) ← PA-small-star-opt(*lout*)

8   *out* ← *sout*

9   *in* ← *in* ∪ *sin*

10  **else**

11   *out* ← LocalCC(*out*)

12 **until** *Convergence*;

 //Computation step

13 **return** CC-Computation(out ∪ in ∪ cc)

**Fig 6:**
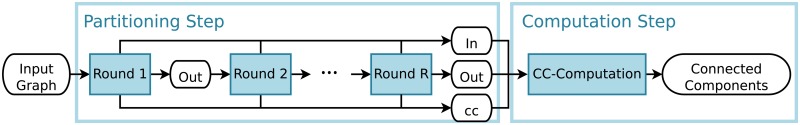
Data-flow in PACC-ef.

Fig 6 shows the data-flow of PACC-ef. In the partitioning step, each round outputs three types of edges: ‘out’, ‘in’, and ‘cc’. The ‘out’ edges is used as input of the next round while ‘in’ and ‘cc’ edges are not. The ‘in’ and ‘cc’ edges are accumulated, and are used for the computation step along with the ‘out’ edges of the final round. CC-Computation of the computation step builds overlapping subgraphs and computes the connected components in each subgraph.

**Algorithm 6**: PA-large-star-opt

 **Map**   : input 〈*u*;*v*〉

1 emit 〈*u*;*v*〉 and 〈*v*;*u*〉

 **Reduce**  : input 〈*u*; Γ(*u*)〉

2 **if** *u* = *m*(*u*) and Γ(*v*) = {*u*} ∀*v* ∈ Γ(*u*) **then**

3  **foreach** *v* ∈ Γ^+^(*u*) **do**

4   emit 〈*v*;*u*〉 to *lcc*

5 **else**

6  **foreach** *v* ∈ Γ^+^(*u*) **do**

7   **if** *v* ≠ *m*_*ξ*(*v*)_(*u*) **then**

8    **if** Γ(*v*) = {*u*} **then**

9     emit 〈*v*;*m*_*ξ*(*v*)_(*u*)〉 to *lin*

10    **else**

11     emit 〈*v*;*m*_*ξ*(*v*)_(*u*)〉 to *lout*

12   **else if** *v* ≠ *m*(*u*) **then**

13    emit 〈*v*;*m*(*u*)〉 to *lout*

**Algorithm 7**: PA-small-star-opt

 **Map**   : input 〈*u*;*v*〉

1 emit 〈*u*;*v*〉 and 〈*v*;*u*〉

 **Reduce**  : input 〈*u*;Γ(*u*)〉

2 **foreach** *v* ∈ Γ^−^(*u*) ∪ {*u*} **do**

3  **if** *v* ≠ *m*_*ξ*(*v*)_(*u*) **then**

4   **if** *v* = *u* and Γ^+^(*u*) = ∅ **then**

5    emit 〈*v*;*m*_*ξ*(*v*)_(*u*)〉 to *sin*

6   **else**

7    emit 〈*v*;*m*_*ξ*(*v*)_(*u*)〉 to *sout*

8  **else if** *v* ≠ *m*(*u*) **then**

9   add a tag to *v* if *v* = *u* and Γ^+^(*u*) = ∅

10   emit 〈*v*;*m*(*u*)〉 to *sout*

The MapReduce version pseudo code of PACC-ef is listed in Algorithm 5. In the partitioning step, PACC-ef alternately performs PA-large-star-opt, and PA-small-star-opt. The pseudo codes of the two operations are listed in Algorithms 6 and 7. For a neighbor *v* of *u*, PA-large-star-opt verifies if Γ(*v*) is {*u*} in constant time by checking *v* has a tag or not, even though we do not actually have the neighbors of *v* in the reduce function of PA-large-star-opt. The tag has been added by PA-small-star-opt of the last round if *v* does not have large neighbors (line 9 of Algorithm 7). Edge (u, v) is ‘cc’ edge if *u* = *m*(*u*) and Γ(*v*) = {*u*}∀*v* ∈ Γ(*u*) because *u* = *m*(*u*) means that *u* does not have small neighbor (i.e., Γ^−^(*u*) = ∅). PA-large-star-opt stores ‘cc’ edges into *lcc* separately (lines 2-4 of Algorithm 6). For edge (u, v), PA-large-star-opt links *v* to *m*_*ξ*(*v*)_(*u*) if *v* ≠ *m*_*ξ*(*v*)_(*u*). If Γ(*v*) = {*u*}, the edge (*v*, *m*_*ξ*(*v*)_(*u*)) is ‘in’ edge, and thus, PA-large-star-opt stores the edge into *lin* separately (line 9 of Algorithm 6). In the reduce function of PA-small-star-opt when the input is 〈*u*; Γ(*u*)〉, *u* is linked to *m*_*ξ*(*u*)_(*u*) if *u* ≠ *m*_*ξ*(*u*)_(*u*). If *u* has no large neighbor, the edge (*u*, *m*_*ξ*(*u*)_(*u*)) is ‘in’ edge, and thus, PA-small-star-opt stores the edge into *sin* separately (line 5 of Algorithm 7).

Decreasing the amount of data to read and write, the edge-filtering quickly drops the number of edges so that the input graph becomes small enough to be processed on a single machine after a few rounds. PACC-ef computes connected component using a single machine with LocalCC when the input size is smaller than a threshold *τ*, instead of running several MapReduce rounds. It saves preparation time for multiple rounds.

### PACC: Sketching for shirinking the input graph size

**Fig 7:**
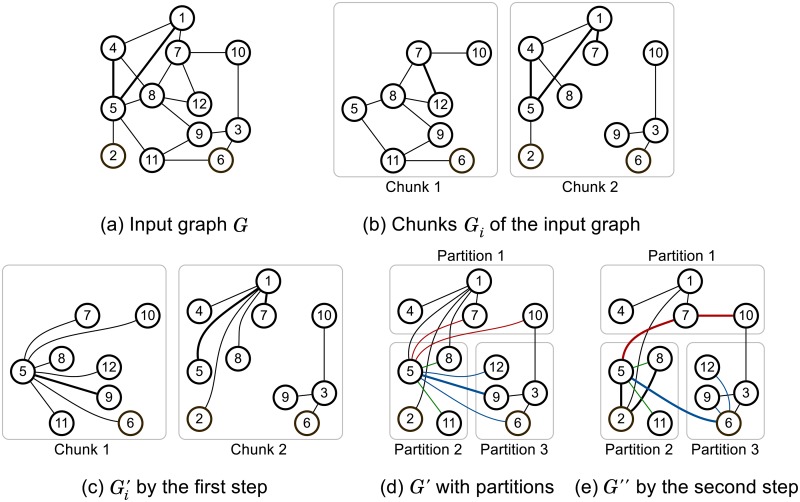
An illustration of sketching. Given *G*, sketching generates new input graph *G*″ via two steps, which has fewer edges than *G*.

The size of input graphs greatly influences the running time of PACC. We propose a sketching method that shrinks the size of an input graph based on the fact that the connectivity of the input graph is preserved even if a part of the graph is replaced by a graph that has the same connectivity. Sketching generates a graph *G*″ with fewer edges than the input graph *G* by two consecutive steps. Then, we use *G*″ as a new input graph of PACC. We explain the method with an example in Fig 7.

*The first step of sketching* reduces the size of the input graph *G* while keeping the connectivity of *G* by computing connected components in edge disjoint subgraphs of *G*. We denote the reduced graph by *G*′. Details of the first step are as follows. The input graph *G* is stored as multiple chunks in a distributed storage, where every edge is included in exactly one of the chunks. Fig 7b shows one possible set of chunks of graph *G* in Fig 7a. We denote the *i*-th chunk and its edge-induced subgraph by *E*_*i*_ and *G*_*i*_ = (*V*_*i*_, *E*_*i*_), respectively, where *V*_*i*_ is the node set of *G*_*i*_, i.e., $\cup_{\left(u,v\right)\in E_{i}}\left\{u,v\right\}$. Sketching computes connected components in each *G*_*i*_ for *i* ∈ {0, ⋯, *C* − 1} where *C* is the number of chunks; that is, the first step outputs $E_{i}^{\prime }=\left\{\left(u,r_{i}\left(u\right)\right)\;|\;u\neq r_{i}\left(u\right),u\in V_{i}\right\}$ for each *i* ∈ {0, ⋯, *C* − 1} where *r*_*i*_(*u*) is the minimum node of the connected component containing *u* in *G*_*i*_. Fig 7c shows the edge induced subgraphs $G_{i}^{\prime }$ of $E_{i}^{\prime }$ computed from *G*_*i*_ in Fig 7b. Then, the entire output of the first step is the union of $E_{i}^{\prime }$, that is, $E^{\prime }=\cup_{i\in \{0,\cdots ,C-1\}}E_{i}^{\prime }$. Fig 7d shows the edge induced subgraph *G*′ of *E*′. Note that the first step is performed in parallel on multiple machines without network communication. The first step of sketching is very similar to the first step of DSP-CC [15], which is an external algorithm. As a result of the first step, the input size is reduced but many edges are concentrated to a few nodes. The edge concentration is not a problem for DSP-CC because DSP-CC is a single machine algorithm, but it causes a load-balancing issue on PACC as PACC is a distributed algorithm. In Fig 7d, for example, most edges are concentrated on node 1 and node 5. The skewness of *G*′ causes a load-balancing issue during the partitioning step of PACC as computation of PA-large-star is concentrated on nodes with a large number of neighbors.

*The second step of sketching* resolves the load-balancing issue by scattering edges concentrated on a small number of nodes while keeping the connectivity of *G*. The second step generates a new input graph *G*″ from *G*′ generated by the first step. For each node *u*, the second step replaces a subset of edges from *u*’s neighbors to *u* with edges between the neighbors. Fig 7e shows the result of the second step on *G*′ in Fig 7d. Let $m_{i}^{+}\left(u\right)$ be the minimum node among the large neighbors [Γ^+^(*u*)]_*i*_ ∪ {*u*} of node *u* that are in partition *i*, that is, $m_{i}^{+}\left(u\right)=\min\left({\left[\Gamma^{+}\left(u\right)\right]}_{i}\cup \left\{u\right\}\right)$. Given node *u* and partition *i*, we define (*u*, *i*)-localization as replacing (*v*, *u*) with $\left(v,m_{i}^{+}\left(u\right)\right)$ for each large neighbor $v\in {\left[\Gamma^{+}\left(u\right)\right]}_{i}\backslash \left\{m_{i}^{+}\left(u\right)\right\}$ in partition *i*. Then, the second step conducts (*u*, *i*)-localization for each node *u* and partition *i*. In Fig 7d, for example, for each partition *i* ∈ {1, 2, 3}, the input edges of (5, *i*)-localization are in red, green, and blue, respectively. The minimum neighbors of node 5, including itself, in each partition are 7, 5, and 6, and thus, other neighbors are linked to one of them as shown in Fig 7e. The two steps of sketching together are implemented with a single MapReduce job and the pseudo code is listed in Algorithm 8.

**Algorithm 8**: Sketching

 **Map**   : input *E*_*i*_

1 **foreach** (*u*, *v*) ∈ LocalCC(*E*_*i*_) **do**

  /* *v* is the minimum node of the connected component including *u* in the *i*-th

   chunk *G*_*i*_. *ξ*(*u*) is the partition containing *u*.      */

2  emit 〈(*ξ*(*u*), *v*);*u*〉

 **Reduce**  : input 〈(*j*, *v*);[Γ^+^(*v*)]_*j*_〉

3 **foreach** *u* ∈ [Γ^+^(*v*)]_*j*_ **do**

4  **if** *u* ≠ *m*_*j*_(*v*) **then**

5   emit 〈*u*;*m*_*j*_(*v*)〉

6  **else**

7   emit 〈*u*;*v*〉

### Analyses

We analyse the correctness and performance of PACC. We first prove the correctness of PACC through Lemmas 2, 3, 4, and Theorem 2.

**Lemma 2**. *PA-large-star and PA-small-star do not break the connectivity of the input graph*.

*Proof*. As proved in Lemmas 1 and 3 of [11], the large-star and the small-star of the alternating algorithm do not break the original connectivity of the initial graph. We show that PA-large-star and PA-small-star have the same connectivity as the large-star and the small-star, respectively. For a node *u* and a large neighbor *v* of *u*, the large-star links *v* to *m*(*u*). PA-large-star links *v* to *m*(*u*) if *v* = *m*_*ξ*(*v*)_(*u*), or connects *v* to *m*(*u*) through *m*_*ξ*(*v*)_(*u*) in the other cases. Thus, PA-large-star also keeps the connectivity of the input graph. For a node *u* and a small neighbor *v* of *u*, the small-star links *u* and *v* to *m*(*u*). Like PA-large-star, PA-small-star connects *v* (or *u*) to *m*(*u*) directly if *v* = *m*_*ξ*(*v*)_(*u*) (or *u* = *m*_*ξ*(*v*)_(*u*)), or via *m*_*ξ*(*v*)_(*u*) in the other cases. Thus, PA-small-star also keeps the connectivity of the input graph.

**Lemma 3**. *The ‘in’ set*, *which is the set of edges filtered out by case 1*, *is a forest (i.e., a set of trees)*.

*Proof*. For edge (*u*, *v*) where *u* < *v* in case 1, *u* is the only neighbor of *v* and the edge is filtered out by PACC-ef. Assume that *v*_*d*_ was a large neighbor of *v* in a previous round, and excluded by case 1; *v* was the only neighbor of *v*_*d*_, meaning that no cycle exists. Thus, the lemma follows.

**Lemma 4**. *The original input graph G and the graph G*″ *generated by the sketching method have the same connected components*.

*Proof*. By the definition of $G_{i}^{\prime }$, *G*_*i*_ and $G_{i}^{\prime }$ have the same connectivity; that is, if two nodes are connected by a path in *G*_*i*_, they are also connected in $G_{i}^{\prime }$. As *G* is the union of *G*_*i*_ and *G*′ is the union of $G_{i}^{\prime }$ for *i* ∈ {0, ⋯, *C* − 1}, *G* and *G*′ also have the same connectivity.

(*u*, *i*)-localization does not change the connectivity of *G*′; after (*u*, *i*)-localization, $m_{i}^{+}\left(u\right)$ is still directly linked to *u*, and *v* that is not $m_{i}^{+}\left(u\right)$ is reachable via $m_{i}^{+}\left(u\right)$. As (*u*, *i*)-localization does not change the connectivity, localization also does not change the connectivity of *G*′. That is, *G*, *G*′, and *G*″ have the same connectivity.

**Theorem 2**. *PACC correctly finds all connected components*.

*Proof*. As proved in Lemma 4, the sketching step of PACC does not change the connectivity of the original input graph. We then show the correctness of PACC by proving the correctness of PACC-ef, which is PACC without sketching.

The partitioning step of PACC-ef outputs three edge sets, ‘in’, ‘cc’, and ‘out’, and the union of the edge sets is the input of CC-Computation. Lemma 3 shows that the ‘in’ set is a forest where a local minimum node in a connected component is the root of a tree. Meanwhile, Lemma 1 shows that all local minimum nodes and the global minimum node of a connected component are linked each other as a star graph in ‘cc’ and ‘out’ of the final round. The two lemmas together show that the union of the three sets is a forest where each tree corresponds to a connected component, and the root is the global minimum node; thus, the forest and the original input graph have the same connectivity. CC-Computation independently computes connected components from *ρ* subgraphs of the forest such that the *i*-th subgraph is the edge-induced subgraph on the edges incident to the nodes in the *i*-th partition. As all local minimum nodes in a connected component are directly linked to the global minimum node in the forest, the nodes of a connected component in *i*-th partition have paths to the global minimum node, which implies the result of CC-Computation is the connected components of the original graph.

The following lemmas present performance bounds of PACC.

**Lemma 5**. *PA-large-star(-opt) and PA-small-star(-opt) never increases the edge number*.

*Proof*. PA-large-star and PA-small-star does not duplicate any edges, and just change each edge with another one. For each edge (*u*, *v*) where *u* < *v*, PA-large-star and PA-small-star change the edge with (*v*, *m*(*u*)) or (*v*, *m*_*ξ*(*v*)_(*u*)).

**Lemma 6**. *Localization does not increase the number of edges*, *that is*, |*E*″| ≤ |*E*′|.

*Proof*. Localization replaces each edge to another edge to scatter the edges concentrated on a few nodes. In other words, the edge number does not increase by localization.

**Lemma 7**. *In PACC-ef*, *the number of input edges into CC-Computation is at most* |*V*| − 1 *where* |*V*| *is the size of node set*.

*Proof*. The input of CC-Computation forms a forest as shown in Theorem 2. Thus, the maximum number of edges in the forest is |*V*| − 1. If the hash function partitions the nodes evenly, the expected edge number in a subgraph is (|*V*| − 1)/*ρ*.

**Lemma 8**. *The number* |*E*″| *of edges generated by sketching is less than or equal to* |*E*| and *C*|*V*|, *where C is the number of chunks*.

*Proof*. Given an edge set *S*, let *G*[*S*] = (*V*[*S*], *S*) be the edge induced subgraph where *V*[*S*] is the node set of *G*[*S*]. Let *r*[*S*](*u*) be the representative node of the connected component containing the node *u* in *G*[*S*]. We define *Z*(*S*) as {(*u*, *r*[*S*](*u*)) | *u* ≠ *r*[*S*](*u*), *u* ∈ *V*[*S*]}. Then, for any nonempty edge set *S*, we first show that

|Z(S)|≤|S|. (1)

By the definition of *Z*(*S*),

|Z(S)|=|V[S]|-|r[S]| (2)

where *r*[*S*] = {*u* ∈ *V*[*S*] | *u* = *r*[*S*](*u*)}. When *S* contains only one edge (*u*, *v*), the inequality holds because *u* and *v* are in the same connected component and one of them must be the representative node. Then, for a nonempty edge set *S* and an edge (*u*, *v*) which is not in *S*, we show that |*Z*(*S* ∪ {(*u*, *v*)})| ≤ |*S* ∪ {(*u*, *v*)}| is satisfied if |*Z*(*S*)| ≤ |*S*|. By (2),

|Z(S∪{(u,v)})|=|V[S∪{(u,v)}]|-|r[S∪{(u,v)}]|

If *u* and *v* both are not in |*V*[*S*]|, |*V*[*S* ∪ {(*u*, *v*)}]| = *V*[*S*] + 2 and |*r*[*S* ∪ {(*u*, *v*)}]| = |*r*[*S*]| + 1 because *u* or *v* is the representative node of the opposite node. Thus,

|Z(S∪{(u,v)})|=|V[S]|-|r[S]|+1=|Z(S)|+1≤|S|+1=|S∪{(u,v)}|

If only one node *u* is in |*V*[*S*]|, |*V*[*S* ∪ {(*u*, *v*)}]| = *V*[*S*] + 1 and |*r*[*S* ∪ {(*u*, *v*)}]| = |*r*[*S*]|; rhus,

|Z(S∪{(u,v)})|≤|S∪{(u,v)}|

If both *u* and *v* are in |*V*[*S*]|, |*V*[*S* ∪ {(*u*, *v*)}]| = |*V*[*S*]| and |*r*[*S* ∪ {(*u*, *v*)}]| = |*r*[*S*]|; accordingly,

|Z(S∪{(u,v)})|=|Z(S)|≤|S|<|S∪{(u,v)}|

Thus, for any nonempty edge set *S*, |*Z*(*S*)| ≤ |*S*| holds.

By the definition of *E*′ and *Z*(*S*),

$$E^{\prime }=\underset{i\in \{0,\cdots ,C-1\}}{\cup }E_{i}^{\prime }=\underset{i\in \{0,\cdots ,C-1\}}{\cup }Z(E_{i})$$

As |*X* ∪ *Y*| ≤ |*X*| + |*Y*| for any sets *X* and *Y*,

$$|E^{\prime }|\leq \sum_{i\in \{0,\cdots ,C-1\}}\left|Z(E_{i})\right|$$

By (1),

$$\sum_{i\in \{0,\cdots ,C-1\}}\left|Z(E_{i})\right|\leq \sum_{i\in \{0,\cdots ,C-1\}}|E_{i}|=\left|E\right|$$

By the definition of *Z*(*E*_*i*_), |*Z*(*E*_*i*_)| ≤ |*V*[*E*_*i*_]| = |*V*_*i*_|, and thus,

$$\begin{array}{c} \begin{array}{cc} \sum_{i\in \{0,\cdots ,C-1\}}\left|Z(E_{i})\right| & \leq \sum_{i\in \{0,\cdots ,C-1\}}|V_{i}|\\ & \leq \sum_{i\in \{0,\cdots ,C-1\}}\left|V\right|=C\left|V\right| \end{array} \end{array}$$ (10)

Finally, by Lemma 6,

$$|E^{\prime \prime }\left|\leq \right|E^{\prime }\left|\leq \min(|E|,C|V|\right)$$

**Lemma 9**. *The expected number of large neighbors of any node after the localization is O*(|*V*|/*ρ* + *ρ*), where *ρ* is the number of partitions.

*Proof*. In the result of (*u*, *i*)-localization, edge $\left(u,m_{i}^{+}\left(u\right)\right)$ is the only edge crossing two partitions if *ξ*(*u*) ≠ *i*, and the nodes of the other edges are in partition *i*. In other words, *u*’s large neighbors in a different partition are generated by only (*u*, *i*)-localization. For each *i* ∈ {0, ⋯, *ρ*−1} \ {*ξ*(*u*)}, (*u*, *i*)-localization can generate an edge crossing partitions *i* and *ξ*(*u*), thus *ρ* − 1 is the maximum number of *u*’s large neighbors not in partition *ξ*(*u*) after the localization. Meanwhile, every node in partition *ξ*(*u*) has a chance to be linked to *u* by an edge after the localization, that is, the maximum number of *u*’s neighbors in partition *ξ*(*u*) is *O*(|*V*|/*ρ*). Thus, the expected number of edges incident to *n* is *O*(|*V*|/*ρ*) + *ρ* − 1 ≤ *O*(|*V*|/*ρ* + *ρ*).

### Implementation

Although we describe PACC using MapReduce primitives for simplicity, PACC can be implemented for various distributed frameworks such as Pregel, Spark, and Pegasus. This section discusses the issues of implementing PACC in Hadoop and Spark, which are the representative distributed computing frameworks.

#### PACC on Hadoop

**Algorithm 9**: PA-large-star-opt combiner

 **Combine**  : input 〈*u*;Γ′(*u*) ⊆ Γ(*u*)〉

1 **foreach** *i* ∈ {0, ⋯, *ρ* − 1} **do**

2  $m_{i}^{\prime }\left(u\right)\leftarrow \infty$

3 **foreach** *v* ∈ Γ′(*u*) **do**

4  **if** *v* > *u* **then**

5   emit 〈*u*;*v*〉

6  **else**

7   $m_{\xi (v)}^{\prime }\left(u\right)\leftarrow \min\left(m_{\xi (v)}^{\prime }\left(u\right),v\right)$

8 **foreach** *i* ∈ {0, ⋯, *ρ* − 1} **do**

9  **if** $m_{i}^{\prime }\left(u\right)\neq \infty$ **then**

10   emit $\langle u;m_{i}^{\prime }\left(u\right)\rangle$

In PA-large-star-opt (Algorithm 6), the map function emits 〈*u*;*v*〉 and 〈*v*;*u*〉 for each edge (*u*, *v*) so that all neighbors of each node are gathered into a reduce operation. Then, for each node *u*, the reduce function links each large neighbor *v* ∈ Γ^+^(*u*) to the global minimum node *m*(*u*) or the local minimum node *m*_*ξ*(*v*)_(*u*) of the partition *ξ*(*v*) of node *v*. We note that small neighbors of *u*, except the local minimum nodes, are not needed in the reduce function. From this, we reduce the amount of shuffled data by using a combine function that excludes small neighbors that cannot be a local minimum node; a combine function in Hadoop conducts a reduce operation in each machine locally, just before the shuffle step. The combine function for the PA-large-star-opt operation is listed in Algorithm 9. The combine function gets a node *u* and a subset Γ′(*u*) of its neighbors as input. For each node *v* ∈ Γ′(*u*), if *v* is a large neighbor of *u*, we emit 〈*u*; *v*〉 (lines 4-5); in other cases, we update $m_{\xi (v)}^{\prime }\left(u\right)$ with *v* (lines 6-7). For each partition *i*, we emit $\langle u;m_{i}^{\prime }\left(u\right)\rangle$ if $m_{i}^{\prime }\left(u\right)$ has been updated at least once (lines 8-10).

**Algorithm 10**: PA-small-star-opt combiner

 **Combine**  : input 〈*u*;Γ′(*u*) ⊆ Γ(*u*)〉

1 *γ* ← ∞

2 **foreach** *v* ∈ Γ′(*u*) **do**

3  **if** *v* < *u* **then**

4   emit 〈*u*;*v*〉

5  **else**

6   *γ* ← *v*

7 **if** *γ* ≠ ∞ **then**

8  emit 〈*u*;*γ*〉

PA-small-star-opt (Algorithm 7), similarly, does not need all large neighbors of each node; one large neighbor is enough to check whether the node has a large neighbor or not (lines 4 and 9 of Algorithm 7). Accordingly, we use a combine function that excludes all but one large neighbor of each node in order to reduce the amount of shuffled data. The combine function for the PA-small-star-opt operation is listed in Algorithm 10. The combine function gets a node *u* and a subset Γ′(*u*) of its neighbors as input. For each node *v* ∈ Γ′(*u*), if *v* is a small neighbor of *u*, we emit 〈*u*; *v*〉 (lines 3-4); in other cases, we update *γ* with *v*. If *γ* has been updated at least once, we emit 〈*u*; *γ*〉 (lines 7-8).

**Fig 8:**
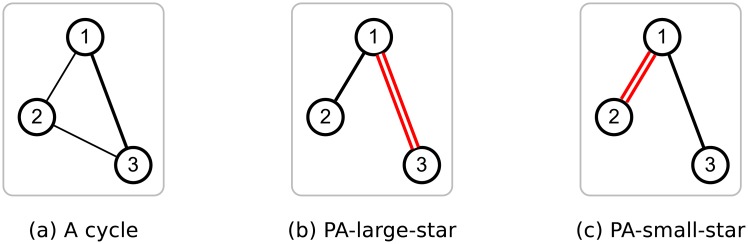
PACC can output duplicate edges from a cycle in both PA-large-star and PA-small-star.

If a graph has a cycle, PACC (and PACC-ef) outputs duplicate edges. PACC can generate plenty of duplicate edges on most real-world graphs which have many cycles. We improve the performance of PACC by eliminating such duplicate edges. Fig 8 shows an example of PACC generating duplicate edges from a cycle. Both PA-large-star and PA-small-star output duplicate edges (1, 3) and (1, 2), respectively. Edge (1, 3) appears twice when performing PA-large-star on node 1 and 2, respectively. Similarly, edge (1, 2) appears twice when performing PA-small-star on node 2 and 3, respectively. PACC removes duplicate edges in the reduce functions of PA-large-star-opt and PA-small-star-opt. Each reduce function gets a node *u* and the neighbors Γ(*u*) of *u* as input. If edge (*u*, *v*) is duplicated, Γ(*u*) contains multiple *v*. That is, removing duplicates in Γ(*u*) means removing duplicate edges. One way to remove duplicates in Γ(*u*) is using a hash table where one slot contains only one element. However, a hash table is effectively slow and requires *O*(Γ(*u*)) space which can exceed the main memory. Instead of using a hash table, we sort and scan Γ(*u*) on external memory.

#### PACC on Spark

Each round of PACC-ef makes three output sets: ‘out’, ‘in’, and ‘cc’. In Spark, one can run three filter operations to make three output sets from a single input set as a filter operation holds only results satisfying a condition and discards the others. This approach, however, has two inefficiency issues. One is that this approach reads the same input data multiple times as each filter operation requires processing entire data, and the other is that all intermediate data (i.e., ‘in’ and ‘cc’ sets) must remain as RDDs until the end of PACC occupying a large amount of memory. We resolve the two issues by storing the output sets except the ‘out’ set into a distributed storage system such as HDFS.

The two star operations of PACC process the neighbors of each node together. Although a groupByKey operation in Spark can gather the neighbors of each node, we use a combination of a partitionBy operation and external sorting on each partition instead of groupByKey because it does not work well with massive data; groupByKey uses memory-based hash tables which assume that data are evenly distributed and fit into memory. Also, even if some data are evenly distributed in memory, hash tables slow down as the data size increases because hash tables frequently require random access accompanying high cache miss rate. Contrary to groupByKey, partitionBy does not use a large hash table because it gathers the neighbors of each node according to the partition, rather than the node. In other words, the neighbors of a node are still mixed up with other nodes’ neighbors in a partition as a set of edges. We gather the neighbors by sorting the edges by the source nodes, then also by the destination nodes to remove duplicate edges. As the size of a partition cannot fit into memory, we use an external sort algorithm. The combination of partitionBy and external sorting significantly increases the scalability and efficiency of PACC on Spark.

## Experiments

This section evaluates PACC. The experimental settings are designed to answer the following questions.

Q1 **Efficacy of Partitioning**. How well does the node-partitioning resolves the load-balancing issue?
Q2 **Efficacy of Edge-Filtering**. How many edges are filtered out by the edge-filtering?
Q3 **Efficacy of Sketching**. How much does sketching shrink the edge number?
Q1 **Scalability**. How well does PACC scale out when the data size and the machine number increase?

In the following, we first introduce datasets and experimental environments, and then, answer the questions with the experimental results.

### Setup

#### Datsets

Table 2 shows the list of the datasets used in our experiments and their sources. Skitter is a computer network, and Patent is a citation relationship graph of US patents. LiveJournal and Friendster are friend-relation graphs in online social media. Twitter is a follower network in the social media of the same name. SubDomain is a hyperlink graph in domain level. YahooWeb is a hyperlink graph in page level. RMAT-*r* is a synthetic graph generated by RMAT model, which is a widely used realistic graph generation model following power-law degree distribution and community structure, where *r* is the recursion level. We use (0.57, 0.19, 0.19, 0.05) for the RMAT parameters (*a*, *b*, *c*, *d*), and use the distributed RMAT generator in TeGViz [26], to generate large graphs. RMAT graphs are for testing scalability of methods so they have various sizes but similar average numbers of neighbors. The target edge numbers for RMAT graphs are 31 457 280, 125 829 120, 503 316 480, 2 013 265 920, and 8 053 063 680 for recursion levels 21, 23, 25, 27, and 29, respectively. We remove all duplicate edges and self-loops beforehand. The numbers of edges in Table 2 are counted except for self-loops and duplicate edges; edges with the same nodes and different direction, e.g., (*u*, *v*) and (*v*, *u*), are also considered as duplicates as we assume all graphs are undirected.

**Table 2:** The summary of datasets.

Dataset	|*V*|	|*E*|	|*E*|/|*V*|	Source
Skitter (SK)	1 696 415	11 095 298	6.54	SNAP^1^
Patent (PT)	3 774 768	16 518 948	4.37	SNAP
LiveJournal (LJ)	4 847 571	68 993 773	14.23	SNAP
Friendster (FS)	65 608 366	1 806 067 135	27.52	SNAP
Twitter (TW)	41 652 230	1 468 365 182	35.25	Kwak et al. [35]^2^
SubDomain (SD)	89 247 739	1 940 007 864	21.73	Webscope^3^
YahooWeb (YW)	720 242 173	6 434 561 035	8.93	Webscope
ClueWeb12 (CW)	6 257 706 595	71 746 553 402	11.47	LemurProject^4^
RMAT-21	731 258	29 519 203	40.36	N/A (Synthetic graphs)
RMAT-23	2 735 400	120 517 935	44.05	
RMAT-25	10 204 129	488 843 429	47.90	
RMAT-27	38 034 673	1 974 122 517	51.90	
RMAT-29	141 509 689	7 947 695 690	56.16	

^1^ http://snap.stanford.edu/data/

^2^ http://an.kaist.ac.kr/traces/WWW2010.html

^3^ http://webscope.sandbox.yahoo.com

^4^ http://www.lemurproject.org/clueweb12

#### Environment

We implement PACC, the alternating algorithm (Alt), and the optimized alternating algorithm (Alt-opt) on Hadoop and Spark. Most experiments are conducted on Hadoop, and a comparison between Hadoop and Spark is presented separately. For PACC, the default values of the threshold *τ* and the number *ρ* of partitions are 20 000 000 and 80, respectively. The parameter *ρ* for Alt and Alt-opt is also 80 unless otherwise noted. The cluster server used for the experiments consists of 20 machines, and each is equipped with Intel Xeon E5-2620v3 CPU (6-cores at 2.40GHz), 32GB RAM, and 4 HDD of 2TB. Hadoop v2.7.3 and Spark v2.2.0 are installed on the cluster with 20 slave machines and one of them also acts as a master. The memory size for a container is 7GB so that one slave can run 4 mappers or 4 reducers concurrently. All running times shown in this paper include all the time to load data and write the results from/to HDFS, the distributed storage used in the experiment.

### Results

#### Efficacy of Partitioning

**Fig 9:**
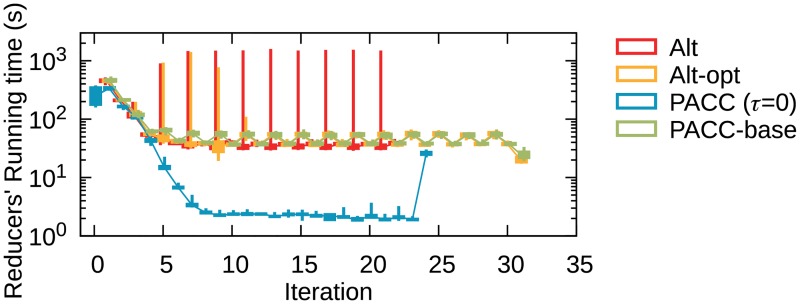
A box plot of reducers’ running time on YahooWeb. PACC, with or without edge filtering and sketching, shows the best load-balancing for every iteration.

Fig 9 presents a box plot of reducers’ running time in each iteration of PACC, Alt, and Alt-opt on YahooWeb; to show the effect of partitioning, we also run PACC only with partitioning, that is, without edge filtering and sketching. We omit the results on other datasets as they have similar trends. The threshold *τ* of PACC is set to 0 to see the running time of entire iterations. The last iterations of PACC and Alt-opt are for the computation step and for cleaning up copied nodes, respectively. The odd-numbered iterations of PACC, Alt, and Alt-opt are of PA-large-star(-opt), large-star, and large-star-opt, respectively. Similarly, the even-numbered iterations of the methods are of PA-small-star(-opt), small-star, and small-star-opt, respectively. The 0-th iteration of PACC is for sketching. The running time of mappers, which has no load-balancing issue, is omitted; accordingly, the initialization time of all methods except complete PACC is omitted because it works only with mappers. The top and the bottom of a box are the third quartile and the first quartile, and a whisker spans the maximum and the minimum running time. Longer whiskers indicate more serious load balancing issues. In the figure, PACC, with or without edge filtering and sketching, shows the best load balancing for every iteration. The running time of PACC with edge filtering and sketching decreases drastically as the graph size decreases with each iteration.

#### Efficacy of edge-filtering

**Fig 10:**
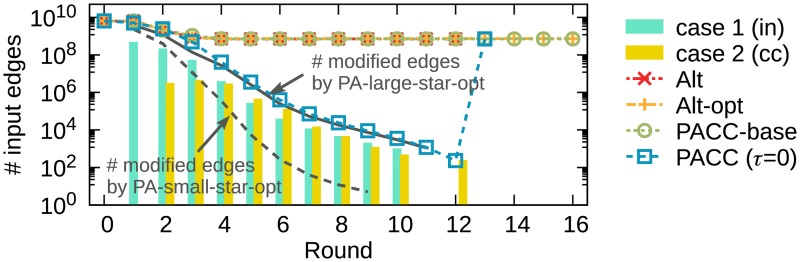
The number of input edges of PACC, Alt, and Alt-opt on YahooWeb at each round. While Alt, Alt-opt, and PACC without edge filtering do not reduce the size of graph below the number of non-root nodes, PACC with edge filtering reduces it to near the lower bound.

Fig 10 shows the input edge size of PACC, Alt, and Alt-opt on YahooWeb in each round. While Alt, Alt-opt, and PACC-base have a limit in reducing the number of input edges, PACC with edge-filtering dramatically reduces the input edge size every round. We depict the numbers of edges modified by PA-large-star-opt and PA-small-star-opt in each round by the solid and dashed lines, respectively, as the lower bound of the number of input edges. The blue line for PACC is very close to the lower bound, showing how effective edge-filtering is. The vertical bars in two colors show the number of added edges to the ‘in’ and ‘out’ sets. The longer the bar, the greater the decrease in the number of input edges in the next round. Edge-filtering also significantly reduces the running time (see Fig 9).

**Fig 11:**
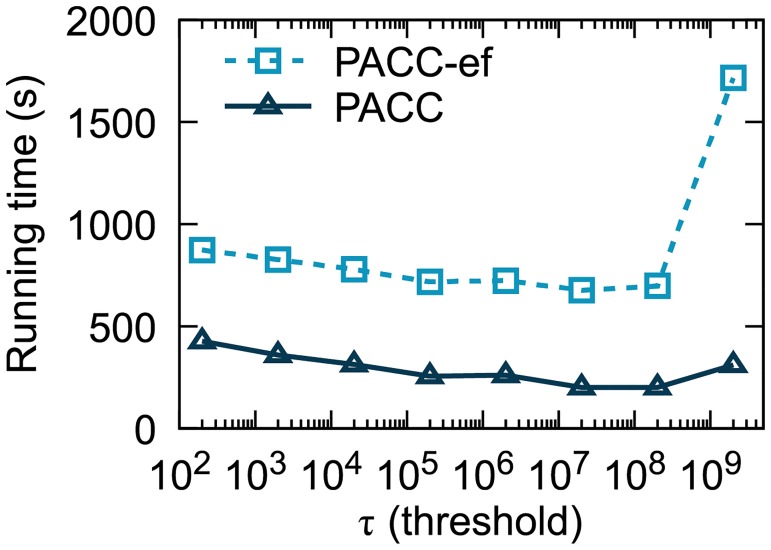
The running time of PACC on various *τ* values. PACC shows the best performance with *τ* = 2 × 10^7^.

Fig 11 shows the running time of PACC on various threshold *τ* on Twitter. With or without sketching, PACC shows the best performance when *τ* is 20 000 000. The running time of PACC-ef sours when *τ* ≥ 2 × 10^9^ because then PACC-ef just runs the single machine algorithm LocalCC after initialization that simply transforms the input files into binary files. On the other hand, the running time of PACC when *τ* ≥ 2 × 10^9^ is not that high compared to the optimal running time even if PACC also runs LocalCC right after sketching, thanks to the reduced input data size by sketching.

#### Efficacy of sketching

**Fig 12:**
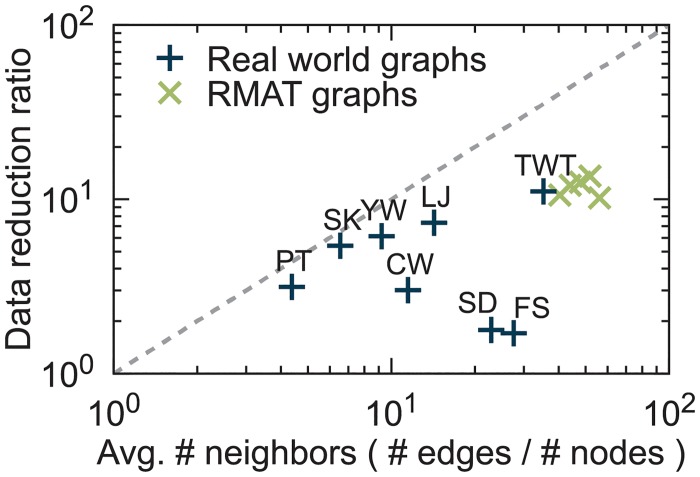
Correlation between the average number of neighbors and the data reduction ratio of sketching.

Sketching boosts the performance of PACC by reducing the input data size. Fig 12 shows how much sketching reduces the size of input data. The data reduction ratio is defined as |*E*|/|*E*″| where |*E*| is the number of edges in the original graph and |*E*″| is the number of edges in the graph generated by sketching. As the upper bound of the data reduction ratio is the average number |*E*|/|*V*| of neighbors, as shown in Lemma 8, we show the correlation between the average number of neighbors and the data reduction ratio of sketching. The dashed line depicts the upper bound. Various data reduction ratios are measured according to the input data; sketching has the best effect on Twitter (data reduction ratio = 11.1) and has the least effect on Friendster (data reduction ratio = 1.7) among real-world graphs. For relatively small datasets such as Patent, Skitter, and LiveJournal, the data reduction ratios are almost close to the upper bound; this is because most edges are included in few chunks. On the other hand, the data reduction ratios are arbitrary for large datasets: Friendster, Twitter, SubDomain, YahooWeb, and ClueWeb12.

#### Scalability

**Fig 13:**
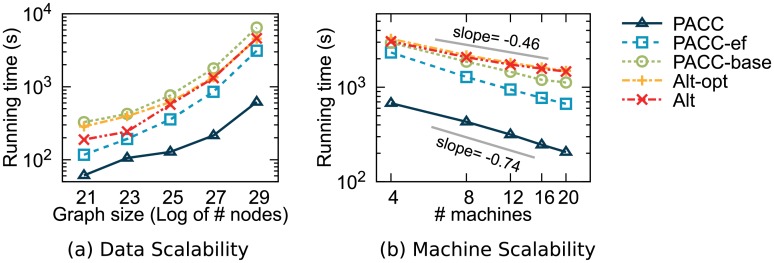
Scalability of PACC, Alt, and Alt-opt. (a) The running time on RMAT graphs with various sizes. PACC exhibits the best scalability as well as the shortest running time for all sizes. (b) The running time with various numbers of machines on Twitter. PACC scales out better than Alt and Alt-opt as the number of machines increases.

Fig 13a shows the running time of PACC, Alt, and Alt-opt on RMAT-S graphs with various sizes. PACC shows the best scalability, which is almost linear, on data size as well as the shortest running time for every RMAT-S graph. Sketching not only reduces the running time of PACC but also increases the scalability on data size; when sketching is included, PACC has a more gradual slope in Fig 13a. This is because sketching has a positive effect on load-balancing. Without sketching, a load balancing problem may occur for the first PA-large-star-opt operation of PACC according to the distribution of input data. After sketching, meanwhile, the maximum number of large neighbors is bounded by Lemma 9, leading to better load-balancing.

Fig 13b shows the running time of PACC, Alt, and Alt-opt in a log scale according to the number of machines on Twitter. The steeper the slope, the better the machine scalability. PACC always shows the fastest performance with the best machine scalability (slope = −0.74). Alt and Alt-opt have the worst machine scalability (slope ≥ −0.48) because of the problem in load balancing; they do not fully utilize all the machines, and only a few machines work hard.

#### Results on real-world graphs

**Fig 14:**
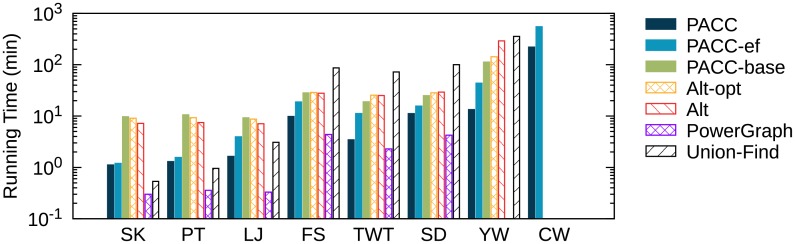
The running time of PACC, Alt, and Alt-opt on Hadoop. PowerGraph and Union-Find are for reference. Missing algorithms for some datasets mean they failed to run on the datasets. Only PACC and PACC-ef succeed in processing ClueWeb12, which consists of 6 billion nodes and 72 billion edges. PACC outperforms Alt-opt showing from 2.9 (on Friendster) to 10.7 (on YahooWeb) times faster performance. Details on the datasets are given in Table 2.

**Fig 15:**
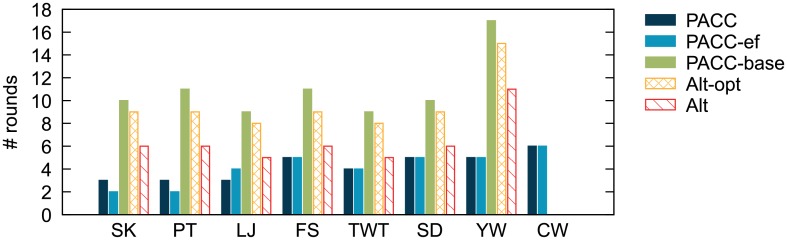
The number of rounds required by PACC, Alt, and Alt-opt on real world graphs in Table 2. PACC requires the smallest number of rounds.

Fig 14 shows the running time of PACC, Alt, and Alt-opt on real world graphs. We also show the running time of PowerGraph, a representative distributed-memory graph processing system, and Union-Find, a representative single machine algorithm, for reference. The proposed algorithm PACC presents the best performance on large graphs, showing up to 10.7 times faster performance than Alt-opt on YahooWeb. Only PACC and PACC-ef succeed in processing ClueWeb12, which is the largest graph used in this paper with 6 billion nodes and 72 billion edges. On small graphs such as Skitter and Patent, all the distributed algorithms on Hadoop have longer running time even than the single machine algorithm; it is because the preparation time for each iteration on Hadoop dominates the entire running time when the graph is small. By the same reason, PowerGraph is faster than all hadoop algorithms, but PowerGraph fails on large graphs (YahooWeb and ClueWeb12) because of an out-of-memory error. For each dataset, the differences in running time between PACC and PACC-ef, and between PACC-ef and PACC-base indicate the effects of sketching and edge-filtering, respectively. The result shows that the decrease in running time by sketching is highly correlated with the data reduction ratio in Fig 12; the running time gaps between PACC and PACC-ef are much bigger on LiveJournal, Twitter, and YahooWeb, whose data reduction ratios are high, than on Friendster and SubDomain, whose data reduction ratios are low. On small graphs, the effect of sketching is insignificant because of the preparation time of Hadoop as mentioned. Meanwhile, edge-filtering tends to be effective on sparse graphs with small average numbers of neighbors such as Skitter, Patent, and YahooWeb, as edge-filtering reduces the round number significantly; the round number required by each algorithm is presented in Fig 15. CC-computation and sketching of PACC are counted as separate rounds, respectively. The number of required round by PACC is smallest compared to the other tested algorithm due to the edge-filtering.

#### Hadoop vs Spark

**Fig 16:**
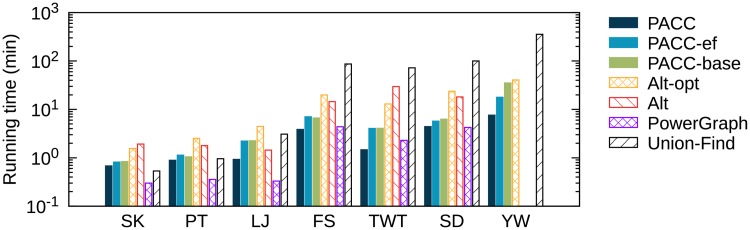
The running time of PACC, Alt, and Alt-opt on Spark. PowerGraph and Union-Find are for reference. Alt and PowerGraph fail on YahooWeb because of out-of-memory errors. ClueWeb12 is omitted because all algorithms fail on it. As in Hadoop, PACC outperforms Alt-opt for every graph in Spark; PACC is 2.3-8.8 times faster than Alt-opt.

**Fig 17:**
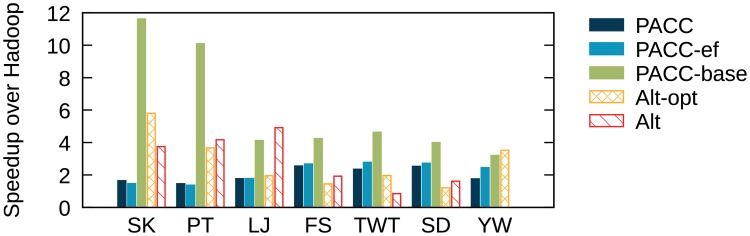
The speedup of Spark over Hadoop on connected component computation. Spark is faster than Hadoop in most cases for all algorithms.

We test PACC, Alt, and Alt-opt on Spark and compare the running times with those on Hadoop. Fig 16 shows the running time of the algorithms on Spark. The running times of PowerGraph and Union-Find are also presented for reference as in Fig 14. On small graphs (Skitter, Patent, and LiveJournal), PowerGraph has the shortest running time. On large graphs (Friendster, Twitter, and SubDomain), PACC shows similar or better performance even than PowerGraph. Compared to Alt-opt, PACC is 2.3-8.8 times faster on all tested graphs. Alt and PowerGraph fail on YahooWeb because of out-of-memory errors. Fig 17 shows speedup over Hadoop on real world graphs. Regardless of algorithms and graphs, the running time on Spark is shorter than that on Hadoop in most cases. The reason is that Spark has shorter preparation time for each iteration than Hadoop does. As Hadoop requires constant preparation time for each iteration, the total preparation time is proportional to the number of rounds. Meanwhile, the total running time increases with the graph size. This means that the preparation time takes a large part of the overall running time if a method requires many rounds while the graph is small; in this case, Spark significantly reduces the total running time, as the preparation time of Spark is much shorter than that of Hadoop. Thus PACC-base, Alt, and Alt-opt, which require many rounds, get a lot of benefit from Spark when the graph is relatively small (Skitter and Patent). Meanwhile, PACC and PACC-ef have few benefits since they require fewer rounds due to edge-filtering. Despite the relatively few benefits from Spark, PACC still shows the best performance on Spark as well as on Hadoop when the graph is large.

## Conclusion

Connected component computation is a fundamental problem in the field of graph mining with various applications such as pattern recognition, graph partitioning, and graph compression. Meanwhile, the existing MapReduce algorithms struggle in the enormity of the graphs with billions of nodes and edges. In this paper, we propose PACC, a fast and scalable algorithm for connected component computation in such large graphs. PACC increases its scalability and performance by three techniques: two step processing of partitioning & computation, edge filtering, and sketching. The two step processing allows PACC to distribute workloads evenly, the edge filtering shrinks the size of intermediate data, and the sketching significantly reduces the input data size. We implement PACC on Hadoop and Spark. The running time of PACC on Spark is shorter than that on Hadoop in most cases; the result is consistent with existing researches that Spark is more suitable for iterative tasks than Hadoop. Regardless of the underlying platform, our experimental results show that PACC shows the best performance on real graphs, with 10.7× faster speed than the most recently proposed MapReduce algorithm.
